# The Role of the Complement System in the Response to Cytotoxic Therapy

**DOI:** 10.1016/j.smim.2024.101927

**Published:** 2025-01-06

**Authors:** Kelly SW. Lee, Qingyang Zhang, Tatsuya Suwa, Heather Clark, Monica M. Olcina

**Affiliations:** Department of Oncology, University of Oxford, Old Road Campus Research Building, Roosevelt Drive, Oxford, OX3 7DQ. United Kingdom

## Abstract

The complement system is increasingly recognised as a key player in tumour progression and response to cancer treatment. Cytotoxic therapies, including chemo- and radiotherapy are standard-of-care for the majority of cancer patients. Cytotoxics have been found to alter expression of complement system proteins and activation of components. Many recent reports highlight the role of local dysregulation of complement proteins in the tumour microenvironment and how targeting such dysregulation can have either anti- or pro-tumouricidal effects depending on several factors including treatment scheduling, the tumour type and its microenvironment characteristics. This review will explore the complex effects of cytotoxic therapy on complement regulation and what lessons can be learnt to identify the most effective way to therapeutically modulate complement system proteins for cancer therapy.

## Introduction

The complement system is an ancient component of innate immunity, crucial in infection control [1, 2]. It consists of over 50 proteins circulating in the plasma and lymph or expressed extra- or intracellularly by cells [3]. Complement activation can occur via three canonical pathways following pathogen- or damage-associated molecular pattern recognition. The classical pathway is activated when C1q binds antigen-bound antibodies, the lectin pathway is induced if mannose-binding lectin, collectin or ficolin interact with pathogenic carbohydrate motifs, and the alternative pathway results following spontaneous C3 hydrolysis [4, 5]. Non-canonical mechanisms involving kallikrein-kinin, coagulation or fibrinolytic proteases can also stimulate complement activity [6, 7]. Regardless of initial differences, all activation pathways converge when C3 convertases form to cleave C3 into C3a and C3b. Membrane-bound C3b enables C5 convertase formation to degrade C5 into C5a and C5b [8]. C5a is a potent anaphylatoxin, mediating inflammation, whilst C5b initiates membrane attack complex assembly to lyse target cells [5]. Regulatory proteins control all pathway stages, restricting immune responses to pathogenic cells [9]. Disruption to spatiotemporal regulation causes excessive complement activity and damages tissues, contributing to inflammatory disease and cancer pathogenesis [5].

**Figure 1:**
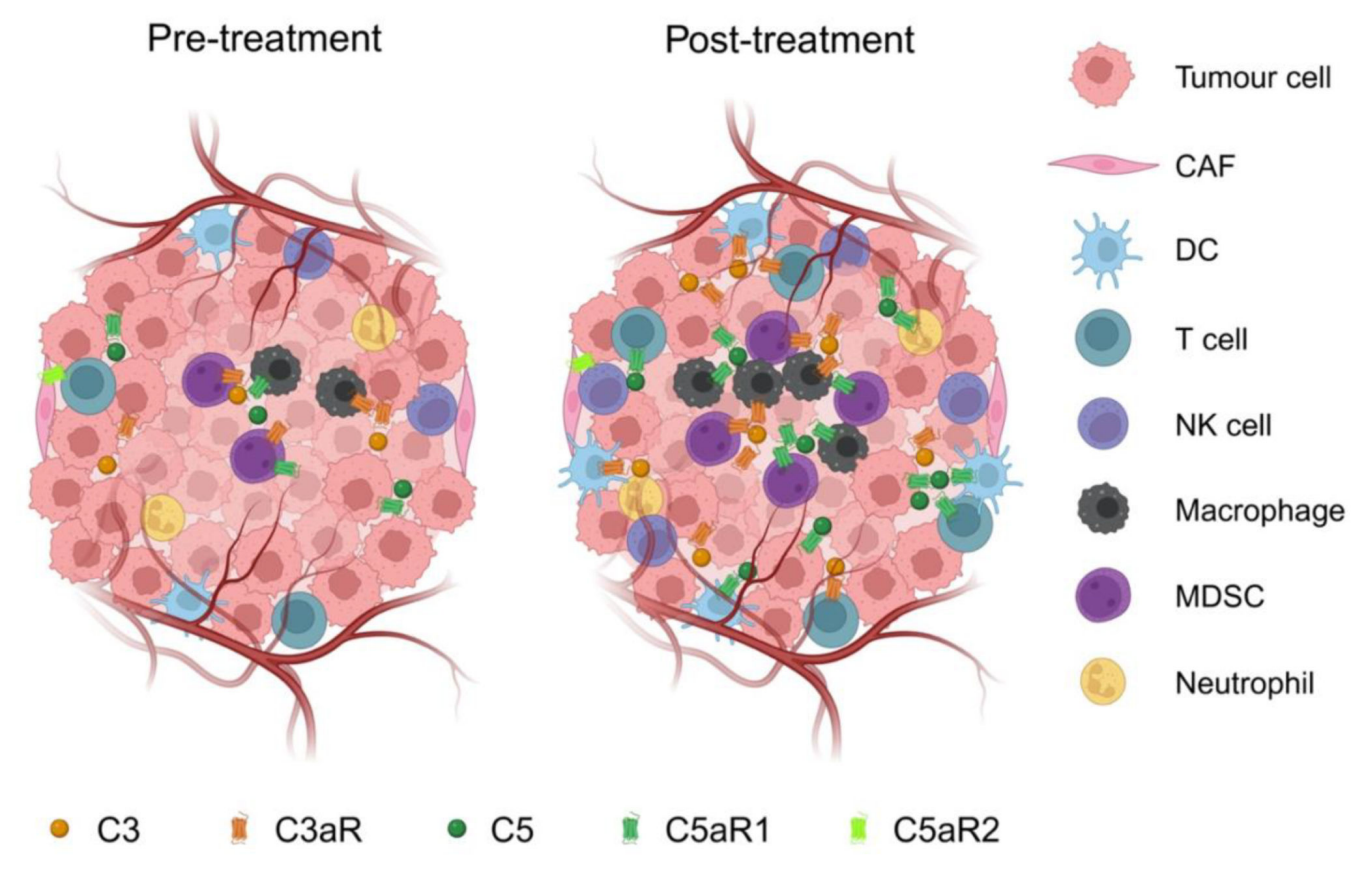
Dysregulation of C3a-C3aR or C5a-C5aR axis in the tumour microenvironment pre- and post-cytotoxic therapy Besides tumour cells, the TME consists of various constituent populations, including fibroblasts, and cells of the lymphoid and myeloid lineage. These cells can express and secrete a variety of complement proteins. Cytotoxic therapies such as chemo- and radiotherapy induce the expression of complement proteins in different cells in the TME as described in the body of the text. Of note, cytotoxic therapies, such as irradiation, also promote tumour infiltration/recruitment of immune and stromal cells expressing complement proteins, which may in turn, alter the overall expression of complement proteins in the TME. Most of the literature has currently focused on the effect of cytotoxic therapies on the C3a-C3aR and C5a-C5aR axes and this is therefore what is represented in this figure. Abbreviation; TME, tumour microenvironment; CAF, Cancer-associated fibroblasts; DC, dendritic cell; TAM, tumor-associated macrophage; MDSC, myeloid-derived suppressor cell; NK cell, natural killer cell; C3aR, C3a receptor; C5aR1, C5a receptor 1, C5aR2, C5a receptor 2.

The tumour microenvironment (TME) consists of multiple cell types, including stromal, immune and tumour cells, that can aberrantly express and secrete complement components (Fig. 1). Uncontrolled complement activity either promotes or hinders tumorigenesis and disease progression in a context-dependent manner [10]. For example, complement dysregulation can mediate immunosuppression, enabling tumour formation and progression [10]. C5a-C5a receptor (C5aR) signalling can recruit myeloid-derived suppressor cells (MDSCs) to the TME to hinder cytotoxic T cell activity via multiple mechanisms, including immunosuppressive cytokine secretion [11–13]. C5a can also recruit or polarise macrophages towards tumour-promoting phenotypes [14–18], whilst both C3a and C5a limit anti-cancer natural killer cell [19] and CD4+ T cell function in some scenarios [20]. Moreover, complement proteins directly affect tumour cell processes, namely replication, metastasis and angiogenesis [21–23]. For example, aberrant C5a and C3 expression can upregulate pro-metastatic transcription factor and matrix metalloproteinase expression, promoting epithelial-mesenchymal transitions and extracellular matrix reorganisation to facilitate tumour cell dissemination [24–28]. As these processes contribute to tumour malignancy, complement dysregulation positively correlates with poor treatment response and disease recurrence in several cancer types [29, 30].

Conversely, some complement-regulated processes have anti-cancer effects [10]. In genetically chimeric mice, endothelial cells were shown to express C3 and C5aR1, with the latter promoting vascular permeabilisation to enable T cell homing [31]. Deletion of C3 or C5aR1 or administration of a C5aR1 inhibitor impaired tumour infiltration of adoptively transferred T cells, increasing tumour cell survival [31]. Bandini *et al*., also detected multiple, larger tumours at earlier timepoints in C3-deleted, Her2/neu transgenic mice with breast carcinoma relative to controls [32]. Here, T regulatory cell (Treg) numbers were significantly increased after C3 deletion and suppressed anti-cancer immune responses.

Targeting complement alone or in combination with standard-of-care cytotoxic treatments is being investigated as an anti-cancer therapeutic strategy [33, 34]. However, the complex, often context-dependent role of complement proteins in the TME at baseline and following treatment with standard-of-care agents currently complicates effective clinical application. Furthermore, previous studies have shown that cytotoxic therapies alter cell distribution in the TME by inducing the recruitment of immune cells (i.e., lymphocytes, myeloid cells and macrophages) into the tumour [35–37]. Since these immune populations can express complement proteins, changes in immune composition in the TME will also alter expression of complement proteins after treatment. Here, we describe the effect of cancer treatment on expression of complement proteins in the TME and overview the role of complement signalling in the response to cytotoxic therapies. A better understanding of the role of complement proteins in the response to standard-of-care cytotoxics can provide insights into complement targeting in the field of oncology.

### Radiotherapy

Radiotherapy is considered standard-of-care for multiple cancer types as primary or adjuvant treatment [38–40]. Radiotherapy is a curative (for localised or isolated metastatic cancer) and palliative (for systemic cancer) treatment. Radiotherapy kills cells by directly damaging DNA or causing indirect damage via water radiolysis and free radical generation [41]. In recent years, radiotherapy has also been shown to have immunomodulatory effects including both increased local tumour-specific immunogenicity and systemic immunogenicity [42, 43]. Previous studies have found that a single dose of 20 Gy or 5 Gy radiation induces upregulation of *C3* transcript in a B16F10-OVA mouse model 4 hours (h) and 24 h after radiation [44]. Similarly, biopsies collected from patients 24-36 h after a single dose of radiotherapy (1.5 or 2 Gy) showed upregulation of complement transcripts such as *C3, C1s, Cfb* and *masp2*. C3 cleavage fragments were also found to be deposited near tumour blood vessels in a rapid and transient manner 24 h after radiotherapy [44]. Surace *et al*., suggested that this is due to IgM binding to necrotic cells following irradiation (single dose, 20 Gy) which in turn activates complement [44]. Furthermore, mice bearing EL4 lymphoma tumours and treated with fractionated radiotherapy (1.5 Gy/day for 4 days) have also been shown to harbour significantly higher C3d complement products compared to non-irradiated tumours [45]. RNA-seq analysis of subcutaneously implanted colorectal tumour organoids, originally derived from *villin*Cre^ER^; *Apc*^fl/fl^; *Kras*^G12D/+^; *Trp53*^fl/fl^ *TgfbrI*^fl/fl^ (AKPT) mice, showed complement cascade genes to be significantly upregulated as early as 4 h after radiation and persisting for up to 7 days [46]. Similar effects were observed in patients, as enriched pathway analysis from rectal adenocarcinoma biopsies showed complement system genes to be significantly upregulated up to 12 weeks following treatment compared to baseline [46]. The elevated level of complement proteins has also been observed in normal brain tissue post-irradiation. For example, radiotherapy induced a 2-fold increase in C1q and C3 level in murine hippocampus, co-localising with IBA1^+^ microglia and GFAP^+^ astrocytic surfaces [47]. Radiotherapy may also trigger upregulation of complement receptors on both the tumour cells and immune cells. C5aR1, a G protein-coupled receptor for complement factor C5a, was found to transiently increase at the protein level in colorectal AKPT murine models after radiation, first predominantly appearing in the epithelial region of the tumour followed by increased expression in Treg, macrophage and neutrophil populations at later time points after radiotherapy [46]. These data could suggest bimodal effects of complement receptors such as C5aR1 in the immune compartment, potentially regulating anti-tumour immunity in the acute setting but later facilitating immune tolerance via immunosuppressive populations such as Tregs or tolerogenic macrophages. Upregulation of *C3, Cfb, C3ar1*, and *C5ar1* mRNA levels in dendritic cells and *C3, C3ar1*, and *C5ar1* in CD8+T cells have also been reported in B16F10-OVA-bearing mice at 24 h and 7 days post radiotherapy [44]. Furthermore, RNA-seq analysis on CD8^+^ T cells from a Lewis lung carcinoma mouse model also showed significantly higher *C5aR1* expression after irradiation [48]. It is important to note that besides direct effects of irradiation on complement-gene expression in different populations within the TME, the increased radiation-induced tumour infiltration of immune cells expressing complement factors could contribute to the changes observed [46].

Whether increased radiation-induced complement activation in the tumour microenvironment is reflected on a systemic level is still unclear, with current studies typically restricted to a handful of complement markers. For example, no significant changes in the plasma levels of C5/C5a were observed following radiotherapy treatment in patients with head and neck cancer [49]. However, sera level of complement may be predictors of neoadjuvant chemoradiotherapy (neo-CRT) as a recent study showed that elevated pre-treatment C3a and C5b-9 levels correlate with poorer outcomes of neo-CRT [50].

In terms of the effect of targeting complement in combination with radiotherapy, reports are mixed, with some studies indicating that targeting C3a/C5a signalling may reduce the effectiveness of radiotherapy while others showing the opposite effects [44–46, 48, 51–53]. Using genetically complement-deficient tumour-bearing mice, Surace *et al*., proposed that C3a and C5a are crucial for radiotherapy efficacy [44]. As mentioned above, radiation induces cancer cell necrosis which may transiently result in increased levels of local C3a and C5a proteins. These anaphylatoxins are crucial for stimulating maturation of tumour-associated dendritic cells and regulating IFN−γ production to sustain the effector functions of tumour-infiltrating CD8+ T cells [44, 54, 55]. In support of these findings, Surace *et al*., indicated that following irradiation, no tumour growth delay was observed in mice lacking C3, C3aR or C5aR1 and implanted with B16F10-OVA ovarian tumour or CT26 colorectal carcinoma. Pharmacological inhibition using a combination of SB290157 (typically reported as a C3aR antagonist) and anti-C5aR1 monoclonal antibody also restricted tumour growth delay following irradiation in B16F10-OVA-bearing mice [44]. Interestingly, a separate study reported that treatment of MOC2 and LY2 tumour bearing mice with SB290157 and C5aR1 receptor antagonist PMX53 did not improve tumour response to irradiation and enhanced tumour growth in the absence of irradiation. The accelerated tumour growth was associated with increased frequency of Tregs [51]. The authors proposed that targeting the C3a/C5a signalling axis drives a phenotypic switch of CD4+ T cells into Tregs. Treg depletion in combination with C3a and C5a receptor inhibition decreased tumour growth compared to the control group [51]. In contrast, in mice with PANC-1 and MIAPaCa-2 pancreatic cancer xenografts, daily administration of antagonist SB290157 resulted in a significant tumour growth delay which was mediated by infiltration of natural killer cells and was independent from CD8+ T cells [53]. It was reported that 14-days treatment with SB290157 had the same effect on tumour growth as a single dose of 5 Gy irradiation [53]. Combining SB290157 and radiation potentiated overall therapeutic benefit with further delay in tumour growth and longer survival [53]. Another benefit of SB290157 was observed in glioblastoma, in which radiotherapy is often offered to patients after surgical resection, but where tumours inevitably progress following treatment [56]. Hypoxia (low oxygen conditions) is well known to contribute to radioresistance in tumours, and a hypoxic tumour microenvironment is associated with upregulated local C3 and C3aR expression in astrocytes and glioblastoma cells [52]. Mice bearing glioblastoma tumours and treated with SB290157 and radiotherapy had longer survival compared to mice treated with radiotherapy alone [52]. This study suggests that complement inhibition could potentially sensitise the most radioresistant tumour cells, enhancing radiation-induced cytotoxicity in radioresistant glioblastoma. Of relevance in interpreting all the studies using SB290157 is the fact that off-target activities have been reported for this compound. For example, SB290157 appears to not only antagonise C3aR but can also act as a partial agonist for C5aR2 [57].

A recent study has also highlighted the importance of understanding the role of C5aR1 in modulating tumour radiation sensitivity. Beach *et al*., showed that administering the C5aR1 antagonist PMX205 improved tumour response following radiation therapy in colorectal subcutaneous tumour models (MC38 and AKPT) [46]. A similar trend was also reported in lung cancer-bearing mice, where combining C5aR1 inhibition (W-54011) with irradiation (3 × 8 Gy) synergistically attenuated tumour growth [48]. Beach *et al*., compared the response to both fractionated and single dose radiotherapy treatment and found that the improved tumour response observed with PMX205 occurred in both the single dose and fractionated irradiation settings in the MC38 model [46]. Interestingly, PMX205 could improve radiation response and enhance tumour cell apoptosis even in tumours with low or no CD8+ T cell infiltration, i.e., an immunosuppressive tumour microenvironment, indicating that this effect is CD8+ T cell independent [46]. Importantly, PMX205 does not promote apoptosis in healthy intestinal epithelium, suggesting that targeting C5aR1 is well-tolerated by non-cancerous cells [46]. Together, these findings suggest that targeting C5aR1 modulates tumour cell radiosensitivity to improve treatment efficacy, even in the absence of CD8+ T-cell infiltration. This is likely to occur at least in part since C5aR1 inhibition attenuates pro-survival signalling to render cancer cells more susceptible to death following radiotherapy [46]. It would be interesting to further investigate whether combinations of complement inhibitors and cytotoxic therapies may enhance other forms of cell death, beyond apoptosis, including whether altered immunogenic cell death programs following complement inhibition may impact downstream anti-tumour inflammatory responses.

Interestingly, using a therapeutic approach aimed at tumour-targeted complement inhibition, Elvington *et al*. demonstrated that C3 inhibition (using recombinant protein CR2-Crry) enhances the therapeutic efficacy of radiotherapy, but in a neutrophil-dependent manner [45]. In this study, in a subcutaneous mouse model of EL4 lymphoma, treatment with recombinant protein CR2-crry and fractionated radiation therapy (15 Gy total in 1.5 Gy fractions administered over 14 days) resulted in more apoptotic cell death following irradiation compared to irradiation or CR2-crry alone [45]. The authors proposed that failure to clear apoptotic cells following complement inhibition resulted in an enhanced inflammatory response, characterised by an early increase in tumour neutrophil numbers and subsequent higher numbers of mature dendritic cells (CD11C+, CD86+, CD80+) and CD8+ T cells in tumours treated with the combination [45].

There are a number of possible explanations for the discrepancies observed between studies indicating that complement inhibition improves tumour radiation response and those indicating the opposite. These could include the difference in cancer types, treatment regime, radiation dose, as well as the type of cells responsible for complement expression in the specific tumour microenvironment context. It should be noted that genetic depletion may have broader systemic implications, such as compensatory mechanisms from other pathways, thus not fully recapitulating pharmacological targeting of C3a-C3aR and C5a-C5aR signalling where inhibition/antagonism occurs within a given discrete timeframe [58]. Furthermore, many studies have shown that in the absence of C3-C3aR or C5a-C5aR signalling, tumour growth in knockout mice is impaired compared to wild type (WT) mice, which complicates the interpretation of the results upon addition of radiotherapy [12, 20, 59–62].

### Chemotherapy

Chemotherapy, the use of cytotoxic compounds to destroy cancerous cells, is also considered standard-of-care (and frequently given in combination with radiotherapy). Triple negative breast cancer is one of the most aggressive subtypes of breast cancer with relatively high incidence of distant metastasis, and lungs being one of the most common metastatic sites [63, 64]. Together with surgical resection, doxorubicin is considered as standard adjuvant treatment but unfortunately, cancer cells often acquire resistance [65]. Mice inoculated with 4T1 breast cancer cells and treated with doxorubicin showed increased C3/C3b deposition in regions near blood vessels 3 days after treatment, and in Tunel^+^ apoptotic tumour cells after 24 days [66]. Cancer-associated fibroblasts (CAF) isolated from mouse lungs bearing early-stage breast cancer metastasis 24 h after the last doxorubicin dose, showed significant upregulation of complement genes across the complement pathway, including *C1ra, C1s, C2, C3, C4a, C5, C7* and *C8a* [63]. Elevated C3 deposits were observed on lung tissue sections from doxorubicin-treated mice compared to vehicle-treated mice and increased C3a levels were also found in lung supernatant following doxorubicin administration [63]. Since C3 is the precursor of the potent chemoattractants C3a and C5a, Monteran *et al*., also showed that CAF-derived chemoattractants facilitate the recruitment of C5aR1-expressing granulocytes and C3aR1-expressing monocytic and granulocytic myeloid-derived suppressor cells (MDSCs), thereby contributing to the formation of an immunosuppressive metastatic niche [63]. However, this may be a doxorubicin specific effect as cisplatin treatment failed to alter C3a deposition [63]. Since MDSCs express complement receptors, Monteran *et al*., blocked receptors C3aR and C5aR1 using SB290157 and PMX53, in combination with doxorubicin treatment post-surgery. The combined treatment of doxorubicin with complement receptor antagonists, induced immune modulation of the tumour metastatic niche and reduced abundance of PD-1+ dysfunctional T cells [63]. Encouragingly, the combination treatment significantly delayed metastatic progression and reduced incidence of advanced metastatic incidence, compared to doxorubicin and complement antagonists as monotherapy [63].

Following doxorubicin-induced tumour cell death, C3 activation was also shown to modulate anti-tumour immune responses through inducing the recruitment of ICOSL expressing B-cells via the CR2 receptor expressed on B-cells. ICOSL^+^ B-cells were proposed to be important for regulating the balance between effector and regulatory T cells. Consequently, B-cells specific CR2 depletion abrogated the anti-tumour effects of doxorubicin [66].

Paclitaxel is another common chemotherapy drug that targets microtubules to arrest cell cycle and promote cancer cell apoptosis [67]. Although the growth of squamous cell carcinoma in mice was not significantly affected by either paclitaxel or C5aR1 antagonist PMX-53 monotherapy, the combination of both treatments was synergistically effective in reducing tumour growth rate [17]. The efficacy of this combination therapy was associated with infiltration of CXCR3+ effector memory CD8+ T cells [17].

In breast and lung cancer, it has been reported that high C5aR2 expression in CAFs is associated with chemoresistance and poor prognosis. Mechanistically, persistent NF-κB activation via C5aR2 in CAFs provides a survival niche for cancer stem cells, promoting tumour formation and chemoresistance. Targeting these C5aR2-expressing CAFs with a neutralising anti-C5aR2 antibody prevented tumour formation and improved tumour chemosensitivity against docetaxel or cisplatin [68].

Clinically, the effects of chemotherapy on complement functions in patients remains unclear. Patients with invasive breast carcinoma showed significantly higher C3 cleavage products in human tumours after chemotherapy [66]. However, when chemotherapy patients’ serum was tested for complement pathway activation via enzyme immunoassay, a large percentage of them failed to form C5b-9 membrane attack complex [69]. Specifically, complement function defects were observed in the alternative pathway (19.1%), the classical pathway (4.3%), or both (42.6%) following chemotherapy protocols such as the ALL-11 (leukemia), the EURAMOS I (osteosarcoma), or the ACNS (medulloblastoma). The effect was particularly prominent following high-dose methotrexate or ifosfamide treatments [69].

## Conclusion

The main goal of cytotoxic treatments such as radiotherapy and chemotherapy is to reduce tumour volume and suppress tumour growth. However, since these cytotoxic treatments also induce local transcription and activation of complement proteins, it is important to establish whether complement proteins act in a pro-tumour or anti-tumour promoting manner in the context of different cytotoxic treatments [12, 20, 44, 48, 59]. Here, we have reviewed the effect of cytotoxics on complement protein expression in the TME and have described the role of these proteins in the response to standard-of-care radio- and chemotherapy (Table 1). Although there is a growing body of studies on targeting the C3-C3aR and C5-C5aR1 axes, the effect of targeting other members of the complement cascade is still mostly unclear. Similarly, it will be important to further explore the effect of cytotoxic treatment on the expression of complement proteins outside of the C3-C3aR and C5-C5aR axes by diverse cells in the TME. This includes understanding whether the subcellular localisation of these proteins is altered by treatment and how these changes may impact tumour response. Underscoring the importance of understanding treatment-induced changes in subcellular localisation of complement-associated proteins, intracellular C4BPA levels have been shown to impact sensitivity to oxaliplatin-induced apoptosis [70]. Furthermore, there is still much to learn regarding the effect of targeting complement with more technologically advanced and emerging radiotherapy modalities such as ultra-high dose rate (FLASH) radiotherapy [71]. Overall, as we think of the context-dependent roles of complement proteins in cancer, it will be important to consider the effect of ‘treatment context’. This will be critical for the most effective clinical translation of complement targeting strategies for the benefit of cancer treatment.

## Figures and Tables

**Table 1:** Anti- or pro- tumour effects of complement-targeted therapy in combination with cytotoxic therapy.

Complement protein	Combination therapy	Affected Cell	Effect	Mechanism	Referrence
C3	Radiotherapy	Neutrophil DC	Anti- tumour	Neutrophil recruitment and increased numbers of mature DCs to support antitumour CD8+ T cells following radiotherapy	Elvington et al., 2014
C3a C5a	Radiotherapy	DC CD8+ T cell	Pro- tumour	Decreased maturation of tumour-associated DCs and IFN-γ production by tumour-infiltrating CD8+T cells	Surace et al., 2015
C3aR	Radiotherapy	NK cell	Anti- tumour	Enhanced tumour NK cell infiltration	Sodji et al., 2022;
C3aR	Radiotherapy	TAM	Anti- tumour	Reduced numbers of tumour “M2-like” macrophages	Rosberg et al., 2024
C3aR C5aR1	Doxorubicin	CAF MDSC	Anti- tumour	Decreased recruitment of MDSCs and reduced PD-1+ dysfunctional T cells in the tumour	Monteran et al., 2022; Danilin et al., 2012; Vadrevu et al., 2014
C5a-C5aR1	Radiotherapy	Cancer cell	Anti- tumour	Attenuated pro-survival signalling to render cancer cells more susceptible to radiation-induced cell death	Beach et al., 2023
C5aR1	Radiotherapy	CD8+ T cell	Anti- tumour	Radiation was associated with enhanced recruitment of CD8+ T cells in which C5aR1 is induced	Yuan et al., 2023
C5aR1	Paclitaxel	CD8+ T cell	Anti- tumour	Increased presence and cytotoxity of CXCR3^+^ effector memory CD8^+^ T cells (dependent on both macrophage and IFNγ)	Medler et al., 2018
C5aR2	Docetaxel or Cisplatin	CAF	Anti- tumour	Reduced NF-kB-dependent cancer stemness, resulting in the reduction of tumour formation and chemotherapy resistance	Su et al., 2018

Abbreviation; DC, Dendritic cell; MDSC, Myeloid-derived suppressor cell; TAM, Tumor associated macrophages; NK cell, natural killer cell; CAF, Cancer-associated fibroblasts; LTB4, leukotriene B4; ROS, reactive oxygen species
